# Supplementary material for: Circular DNA elements of chromosomal origin are common in healthy human somatic tissue
Source: Nat Commun. 2018 Mar 14;9:1069. doi: 10.1038/s41467-018-03369-8 (PMC5852086; doi:10.1038/s41467-018-03369-8)
Supplement: Supplementary file 1 — Supplementary Information [file 41467_2018_3369_MOESM1_ESM.pdf]

Supplementary Information

Møller et al., 2018:

Circular DNA elements of chromosomal origin are common in healthy human somatic tissue

Supplementary Note 1. Terminology and nomenclature for extrachromosomal circular DNA. The existence of eccDNAs in higher eukaryotes has been known for more than half a century[1]. Nonetheless, a common nomenclature has not been established, leading to a range of terminologies. For instance, our preferred eccDNA abbreviation covers other entities such as amplisomes[2], circular extrachromosomal DNA[3], covalently closed DNA circles[4], cryptic circular DNA[5], double minutes[1], episomes[6,7], microDNA[8], minicircles[9], micro chromosomes[10], ring chromosomes[11,12] and small polydispersed circular DNA[13,14] (for review see[15]). We used the name extrachromosomal circular DNA, eccDNA, as the collective term for these elements and encourage the unifying nomenclature of eccDNA to describe all circular DNA elements from eukaryotes to avoid confusion.

The large number of eccDNAs recorded in the present study calls for an eccDNA nomenclature. Brackets around a the name of a genetic element, [*GENE X*], are conventionally applied to describe genetic elements with non-Mendelian inherence in yeasts and bacteria, and are currently used to describe eccDNA from yeast[16-19]. We furthermore used a superscripted "circle," [$GENE\ X^{circle}$], to indicate the circular nature of eccDNA.

Supplementary Fig. 1

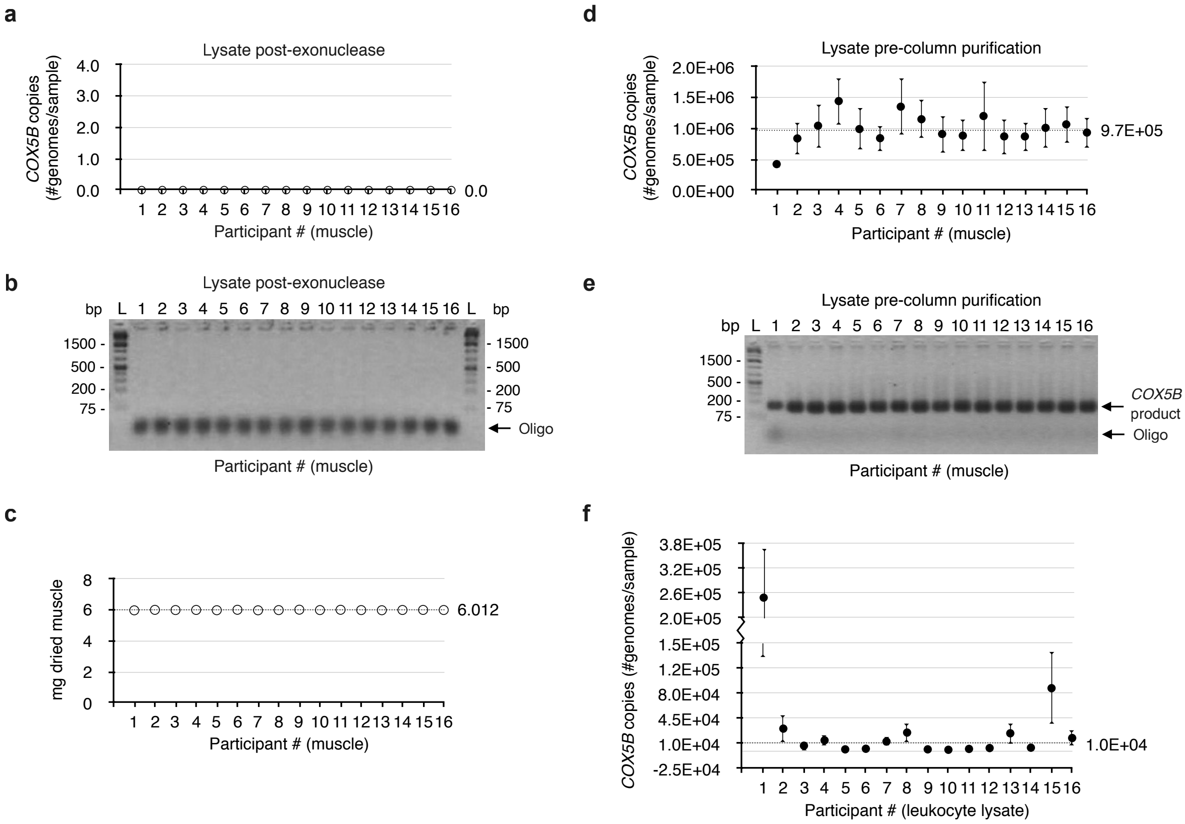

Supplementary Figure 1. Quantification of linear DNA. a, *COX5B* copies quantified after 5-day exonuclease treatment of muscle samples after column purification (Circle-Seq method). **b**, Representative gel-image from qPCR experiment in **a**. **c**, Dried muscle fibers in mg from healthy participants (T1 to T16, n =16), used as input for the Circle-Seq method (median 6.012 mg ± 0.012 mg). **d**, Genomes per muscle lysate sample estimated from *COX5B* copies (median 9.7 x 10^5 ± 2.9 x 10^5) before column purification (Circle-Seq method). **e**, Gel image of a representative quantitative PCR (qPCR) experiment from **d**. **f**, *COX5B* copies per leukocyte-enriched blood lysate sample (B1 to B16, n = 16, median 10^4 ± 6.1 x 10^3) pre-column purification (Circle-Seq). All qPCR reactions were performed in quadruplicate and averages from each experiment were used to calculate displayed standard deviations between a minimum of two independent qPCR experiments.

Supplementary Fig. 2

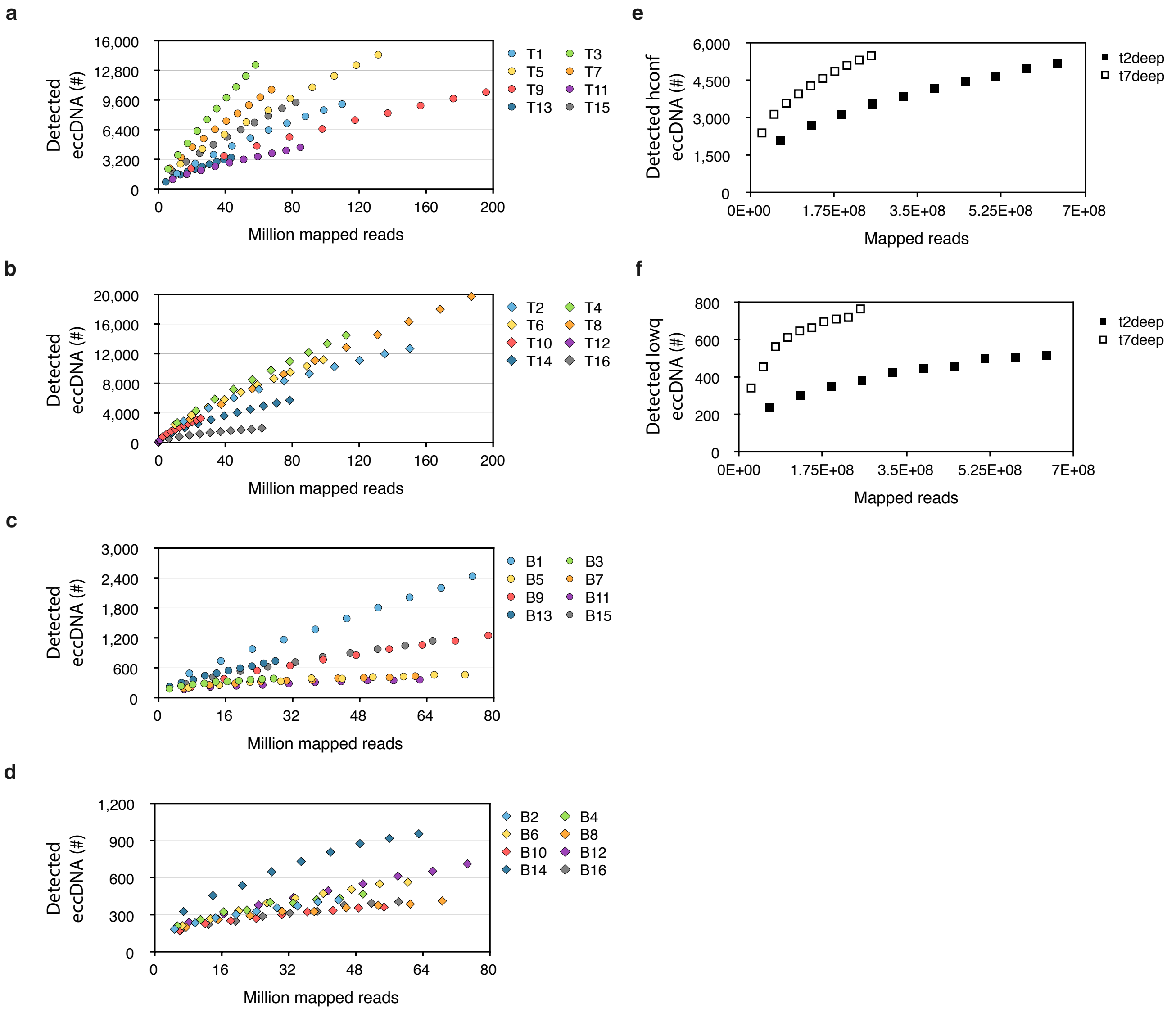

Supplementary Figure 2. Decimation plots of eccDNA counts from samples of skeletal muscle and leukocytes as function of million mapped reads derived from sequencing **a-b**, muscle-derived eccDNA (T1-T16, n =16) and **c-d**, blood-derived eccDNA (B1-B16, n = 16). **a** and **c**, Participants with a sedentary lifestyle; b and d, participants with a physically active lifestyle. **e**, Comparison of detected eccDNA counts at high confidence (hconf) for in-depth sequenced samples T2 and T7 and **f**, eccDNA recorded with less confidence (lowq).

Supplementary Fig. 3

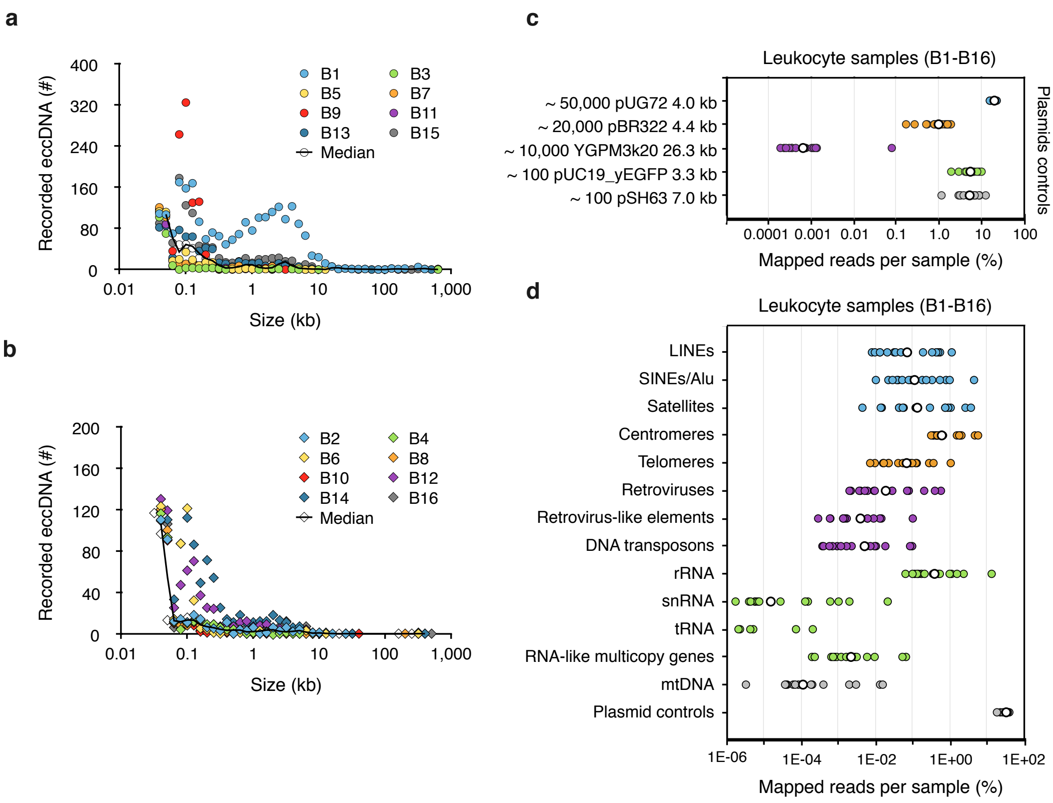

Supplementary Figure 3. Leukocyte-derived eccDNA size and read distribution on plasmids and repetitive elements. Number of leukocyte-derived eccDNAs relative to size in kb in 10^4 leukocytes from each of the 16 participants and median from **a**, men with a sedentary lifestyle (n = 8) and **b**, physically active men (n = 8). EccDNA kb sizes are in bins of 0.1 log-fold changes. **c**, Reads mapped to plasmid controls added to leukocyte samples before eccDNA purification at indicated copy numbers; median, white circles. **d**, Reads mapped to repetitive regions for leukocyte-derived eccDNA samples (B1-B16) as percentage of total mapped reads; median, white circles (n = 16). Satellites, blue (n = 15) and group of non-LTR retrotransposons (n>100, LINE and SINE/Alu); centromere and telomeres, orange; LTR retrotransposons and transposable elements (n>200), purple; RNA genes encoding either ribosomal, small nuclear, transport or multicopy RNA-like repeats (n = 26), green; mitochondrial DNA (mtDNA) and plasmid controls, gray (n=6).

Supplementary Fig. 4

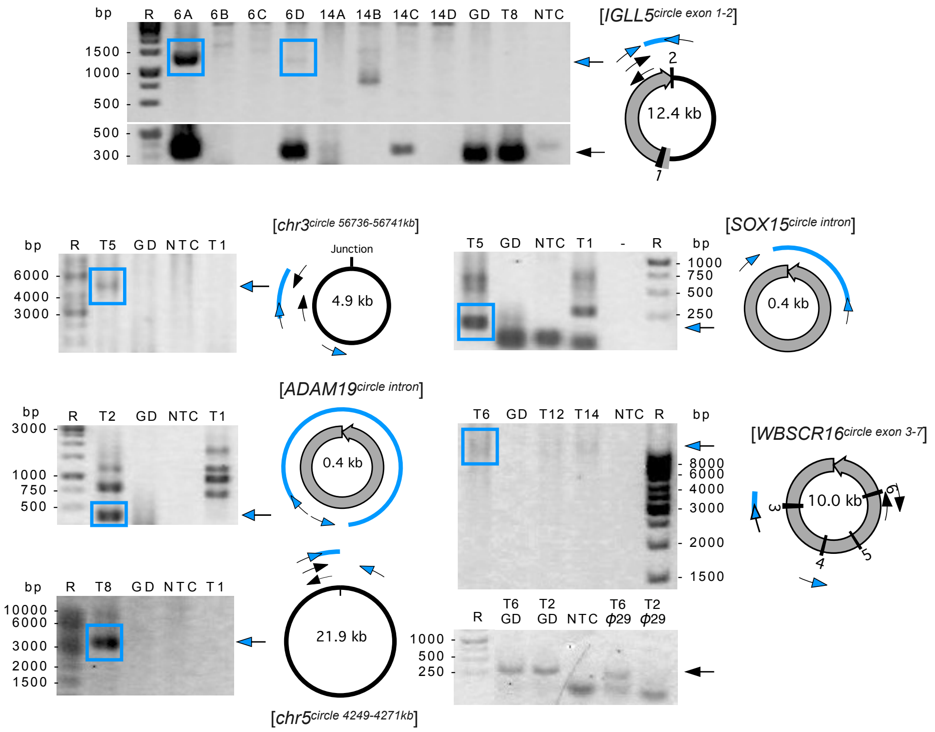

Supplementary Figure 4. Gel images representing a validated subset (n = 6) of recorded eccDNAs by outward PCR (blue arrows), inward PCR (black arrows), gel electrophoresis and Sanger sequencing. EccDNAs are named according to gene content; black boxes, exons. Template: phi29 (ϕ) amplified eccDNA from muscle T1, T2, T5, T6, T8, T12, T14; leukocyte B6A-C, B14A-C; GD, genomic DNA; NTC, nontemplate control. Sanger sequenced PCR products were aligned to eccDNA and the resultant sequence shown as blue lines.

Supplementary Fig. 5

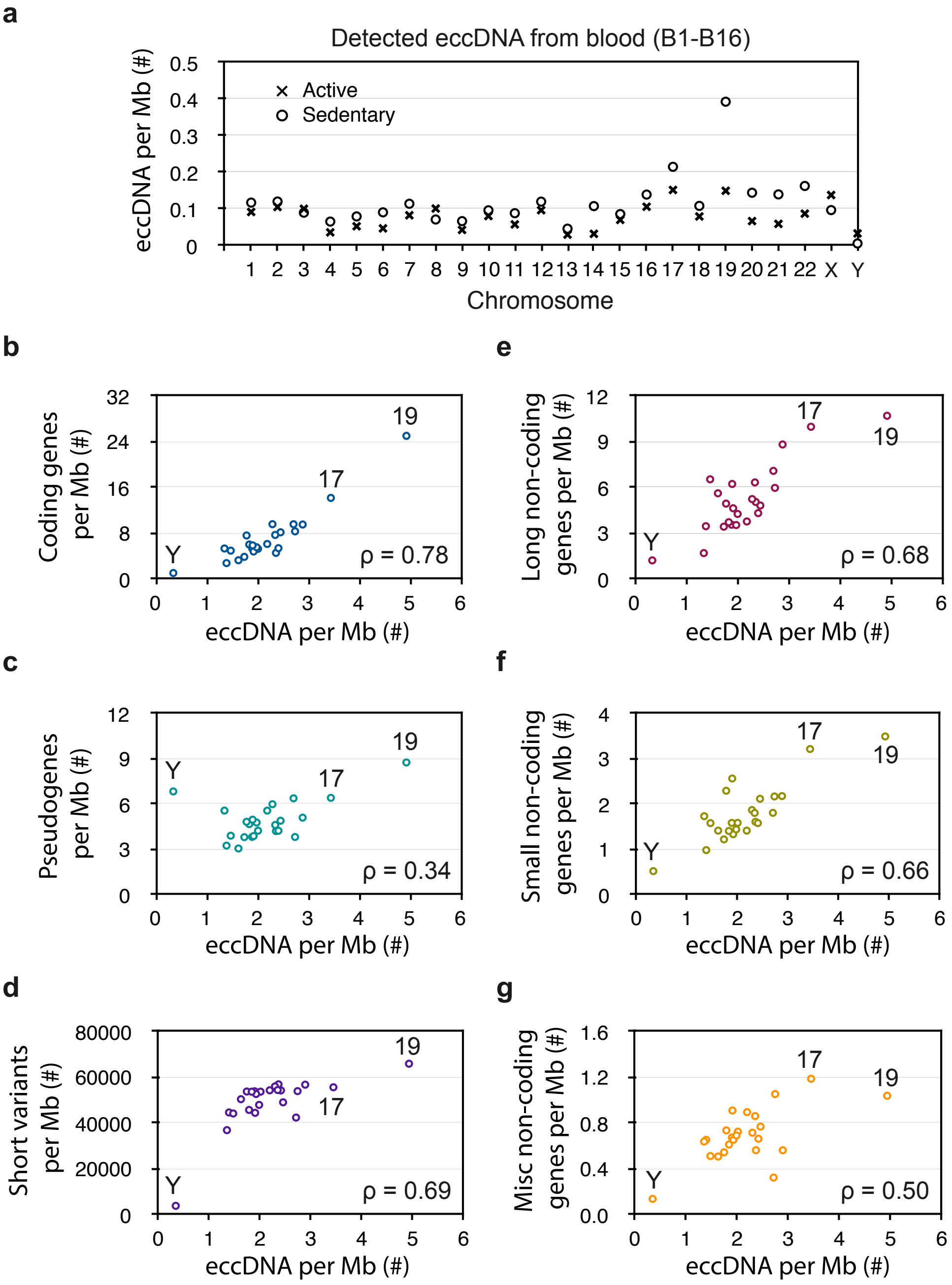

Supplementary Figure 5. EccDNA frequency relative to chromosomes and chromosomal features. a, Leukocyte-derived eccDNA counts per Mb from active (x, n = 8) and sedentary men (o, n = 8) relative to each chromosome. **b-g**, Correlation between eccDNA per Mb (average, n = 16) and **b**, coding genes per Mb, **c**, pseudogenes per Mb, **d**, short variants per Mb, **e**, long noncoding genes per Mb, **f**, small noncoding genes per Mb, and **g**, miscellaneous types of noncoding genes per Mb. Rho (ρ), Spearman rank correlation coefficient; Y, 17 and 19, marked human chromosomes.

Supplementary Fig. 6

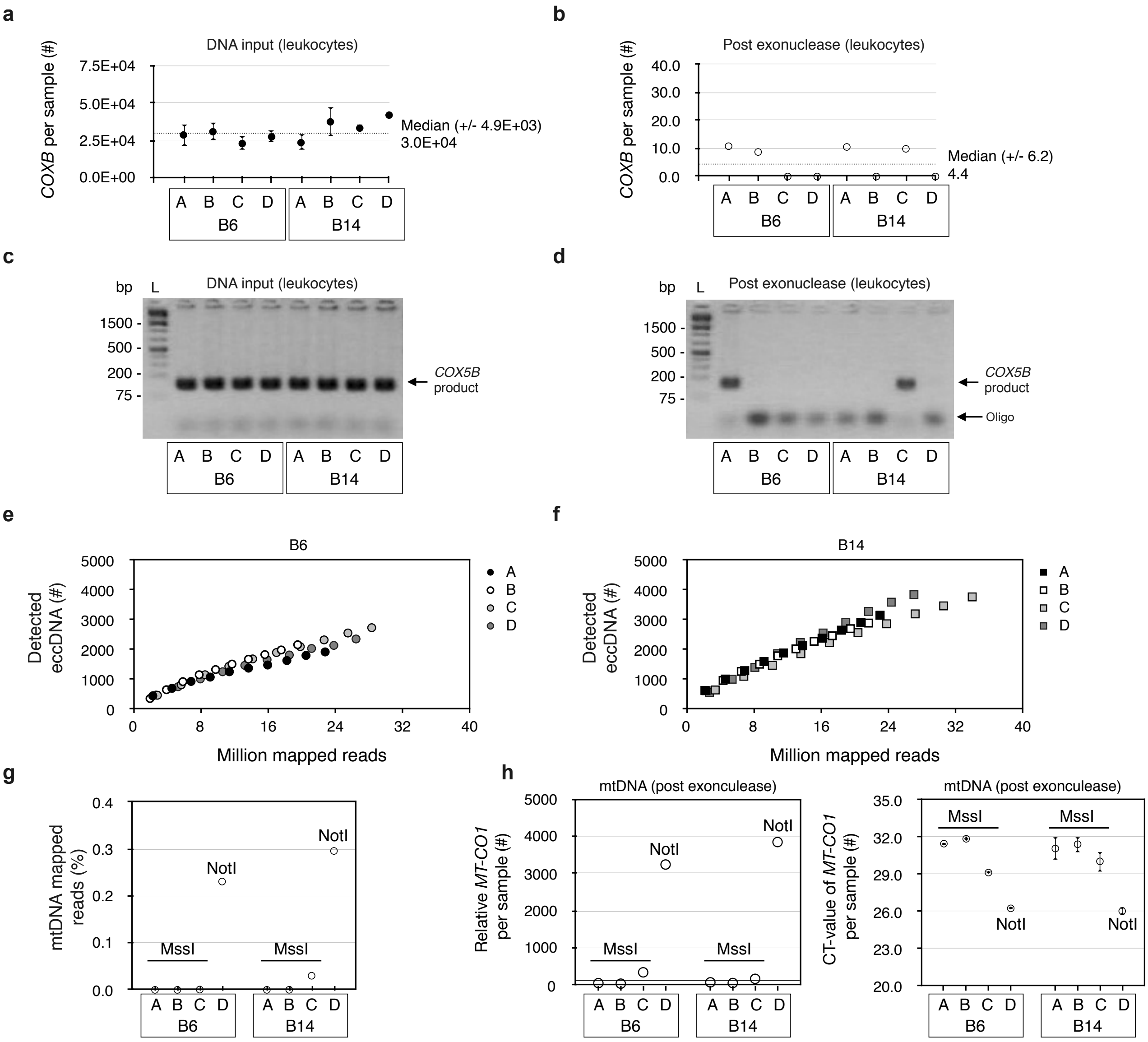

Supplementary Figure 6. Quantification of DNA concentration and eccDNA count relative to mapped reads. a, Quantitative PCR (qPCR) assessment of the number of *COX5B* copies per leukocyte lysate (n = 4, A-D) from participant 6 and participant 14 before column purification by Circle-Seq (median $3.0 \times 10^4 \pm 4.9 \times 10^3$). **b**, Quantified *COX5B* copies after treatment with 210 units exonuclease (median 4.4 ± 6.2). **c-d**, Representative gel images after qPCR experiments from **a** and **b**, respectively, showing size-specific *COX5B* products and oligos. All qPCR reactions were performed in quadruplicate and a minimum of two independent experiments were used to calculate average standard deviations. **e-f**, Decimation plots of eccDNA counts in leukocytes as function of million mapped reads in **e**, B6A-C and **f**, B14A-C. **g**, Circle-Seq detection of mitochondrial DNA (mtDNA) in *Not*I-treated samples (B6D and B14D) but not in *Mss*I-treated samples B6A-C and B14A-B (few % mtDNA reads in B14C). **h**, QPCR quantification of the *MT-CO1* gene on mtDNA for sample B6A-D and B14A-D; left, relative *MT-CO1* copy numbers between samples; right, qPCR CT-values.

Supplementary Fig. 7

a

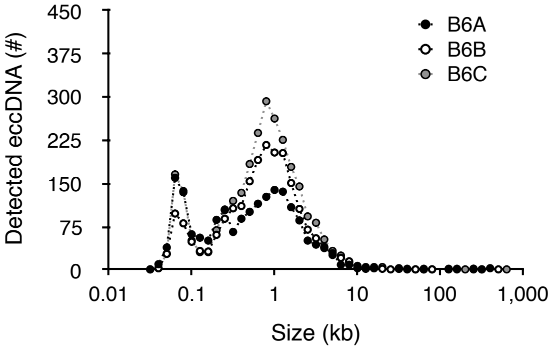

b

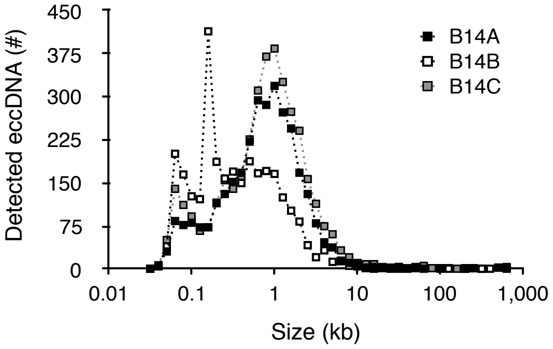

c

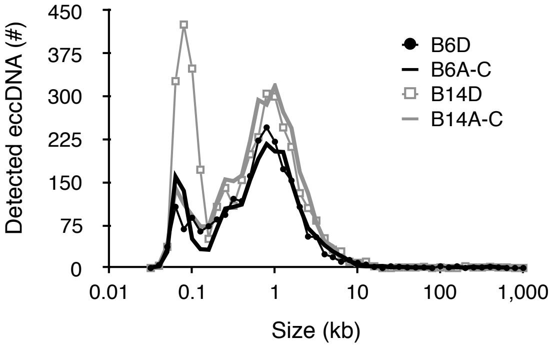

Supplementary Figure 7. Size distribution of eccDNAs in leukocytes. Detected leukocyte-derived eccDNAs relative to size in kb in two sets of triplicate samples (3 x 10^4 leukocytes/sample) from **a**, participant 6 and **b**, participant 14. EccDNA kb-sizes are in bins of 0.1 log-fold changes. **c**, Median eccDNA counts of B6A-C and B14A-C (treated with *Mss*I endonuclease) with comparison to B6D and B14D (treated with *Not*I endonuclease).

Supplementary Fig. 8

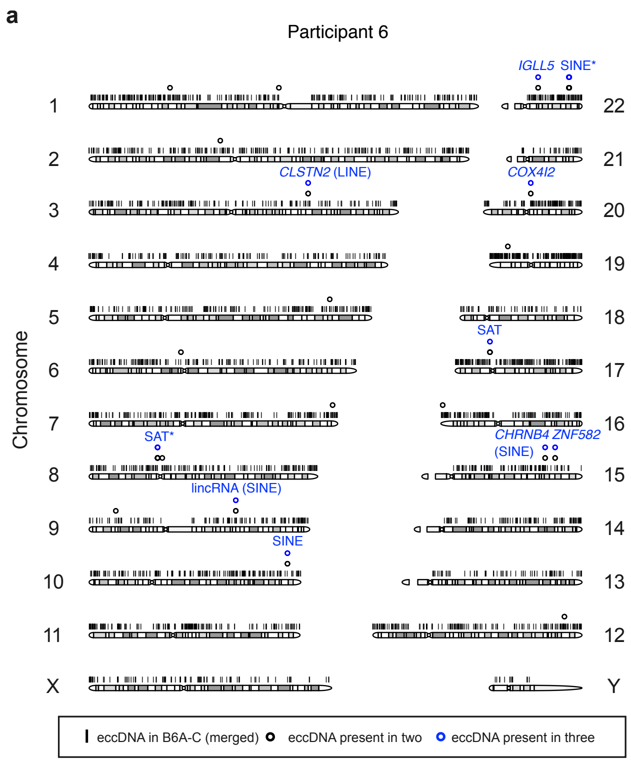

Supplementary Figure 8. EccDNA from leukocytes from participant 6 and 14. Human genomic map of all detected eccDNAs in **a**, B6A-C (n = 3) and **b**, B14A-C (n = 3). **a-b**, EccDNA detected once, black lines; twice (≥90% overlap), black circles; three times (≥90% overlap), blue circles. Classes: SAT, satellites (ALR/alpha); LTR, long terminal repeat (THE1C); SINE (Alu family); lincRNA, DNA encoding long intergenic noncoding RNA; I, intergenic. * = several detected eccDNAs of same class.

Supplementary tables with legends

Parameter	Inactive (n=8)	Active (n=8)
Age (years)	62.8 ± 1.3	62.1 ± 1.4
Height (cm)	176 ± 9	180 ± 8
Body weight (kg)	82.9 ± 14	82.3 ± 12
Body fat (%)	30.5 ± 7.9	21.9 ± 4.2 *
Incremental cycling test duration (min)	13.1 ± 3.6	20.8 ± 5.1 **
Incremental cycling test (wattmax)	188 ± 36	266 ± 51 **
Total blood cholesterol (mmol/L)	5.73 ± 1.16	5.44 ± 0.88
HDL cholesterol (mmol/L)	1.50 ± 0.47	1.60 ± 0.46
LDL cholesterol (mmol/L)	3.54 ± 0.81	3.41 ± 0.78
Total cholesterol/HDL cholesterol	4.17 ± 1.39	3.69 ± 1.32
HDL cholesterol/LDL cholesterol	0.44 ± 0.18	0.50 ± 0.21
Total leukocytes (10^9 cells/L)	7.1 ± 1.7	6.8 ± 1.8
C-Reactive Protein, inflammation test (mg/L)	1.7 ± 0.8	1.0 ± 0.8
Testosterone (ng/dL)	12.4 ± 4.1	12.7 ± 6.4
Protein carbonylation (nmol/mg protein)	1.94 ± 0.58	1.37 ± 0.28 *

Supplementary Table 1. Physiological data from healthy human donors. Physiological parameters of healthy men with a lifelong sedentary or lifelong physically active lifestyle (each group n = 8), showing mean ± standard deviation. Cycling endurance, percent body fat, protein carbonylation were significantly different between the two groups (two-tailed t-test, p-values * < 0.05; ** < 0.005). Selection of men for the two groups was based on questionnaires. Physically inactive men had a lifelong sedentary lifestyle with physical activity once per week at most throughout their life. Physically active men had exercised throughout their life three times per week or more.

Read support of eccDNA detection	TISSUE (T1-T16, n = 16)	LEUKOCYTES (B1-B16, n = 16)	NA12878 (Platinum genome)
% eccDNAs with ≥ 1 soft-clipped read (# eccDNA)	38.9 (53890)	22.7 (2325)	0.75 (54)
% eccDNAs with ≥ 1 discordant paired-end read	78.8 (109241)	85.7 (8764)	99.25 (7144)
% eccDNAs with ≥ 1 split read (# eccDNA)	45.3 (62763)	27.4 (2799)	0.0 (0)
% total (# total)	100 (138681)	100 (10228)	100 (7198)

Ranked eccDNA detection	TISSUE (T1-T16, n = 16)	LEUKOCYTES (B1-B16, n = 16)	NA12878 (Platinum genome)
% hconf (# hconf)	31.7 (43960)	61.1 (6253)	17.4 (1255)
% conf (# conf)	58.5 (81066)	31.2 (3191)	72.9 (5246)
% lowq (# lowq)	9.8 (13655)	7.7 (784)	9.7 (697)
% total (# total)	100 (138681)	100 (10228)	100 (7198)

EccDNA detections in repeat-masked regions	TISSUE (T1-T16, n = 16)	LEUKOCYTES (B1-B16, n = 16)	NA12878 (Platinum genome)
% hconf (# hconf)	26.9 (37301)	58.6 (5990)	16.1 (1157)
% conf (# conf)	45.9 (63591)	26.7 (2730)	67.0 (4826)
% lowq (# lowq)	9.3 (12880)	7.0 (716)	9.4 (678)
% total (# total)	82.0 (113772)	92.3 (9436)	92.5 (6661)

Supplementary Table 2. Detected eccDNAs in tissue, blood and the Platinum data set NA12878.

EccDNA gene content	EccDNA (kb)	Sample	Chr	Start (bp)	End (bp)	Detection confi-dence	Outward oligo's	Outward product (kb)	Inward oligo's	Inward product (kb)
TTN	35.4	T5	2	179591537	179626959	hconf	5' ACCTTTGCTTCCCGAACCTACG 3'; 5' GCTAATGAAGTCGGCAAGTGTGG 3'	13.8	5' TGAACCCTGCCCACAAAGAGC 3'; 5' AGTGCTTAGTTCGTGGCTCTCC 3'	0.9
TTN	23.9	T6	2	179597590	179621459	hconf	5' ACCTTTGCTTCCCGAACCTACG 3'; 5' GCTAATGAAGTCGGCAAGTGTGG 3'	2.3	5' TGAACCCTGCCCACAAAGAGC 3'; 5' AGTGCTTAGTTCGTGGCTCTCC 3'	0.9
ARHGEF3	4.9	T5	3	56735996	56740870	hconf	5' CCGAGATTTGGGGAGCAGAAGG 3'; 5' TACTGCGGGGAGAGGGAAATGG 3'	4.4	5' CCGAGATTTGGGGAGCAGAAGG 3'; 5' GCCATGATTGCACCACCATACTCC 3'	0.8
SLC2A9	4	T8	4	9992579	9996591	hconf	5' GCAAGCAGAGGACAGAGGATGC 3'; 5' CAGGAACCCAAAGCAAACTGGC 3'	3.1	5' GCAAGCAGAGGACAGAGGATGC 3'; 5' GACACCTCACTTCTCAGCCAGC 3'	1
SLC2A9	10.7	T15	4	9987708	9998430	hconf	5' GCAAGCAGAGGACAGAGGATGC 3'; 5' CAGGAACCCAAAGCAAACTGGC 3'	9.8	5' GCAAGCAGAGGACAGAGGATGC 3'; 5' GACACCTCACTTCTCAGCCAGC 3'	1
ADAM19	0.4	T2	5	156981963	156982366	hconf	5' GGTCCCCATCTGCCAAATAG 3'; 5' GGTGTGGAGCAGTGTGACTTGC 3'	0.4	-	-
MYH1	20.1	T2	17	10393080	10413182	hconf	5' CTCCTCCTCATACTGTTCCCGC 3'; 5' TTAGTTTGCTGGATTGTGCCCC 3'	13.1	5' CTCCTCCTCATACTGTTCCCGC 3'; 5' TTTGTTGTCACCCATGCCCTCC 3'	0.5
SOX15	0.4	T5	17	7492557	7492969	hconf	5' GTCCCGAAGATGAGGAGGCAGC 3'; 5' CGACTACAAGTACCGGCCTCG 3'	0.2	-	-
**Intergenic region**	21.9	T8	5	4248712	4270566	hconf	5' ATGAAGTGGGAGGAGCAGGACC 3'; 5' TTTCCCTCTGGTCACAAGCTGG 3'	3.3	5' ATGAAGTGGGAGGAGCAGGACC 3'; 5' TGAATCTCAGCACCACCTCCTCC 3'	0.3
DAZ4	2.4	T14	Y	27015112	27017510	hconf	5' GGGAGCAAAGGGATTATGTTGTCC 3'; 5' GGGAGTTGGCAATGTGGAAACC 3'	2.2	5' GGGAGTTGGCAATGTGGAAACC 3'; 5' TGGTGTCCCCTTCTTCATTTGTGG 3'	0.3
WBSCR17	9.3	T8	7	70850120	70859421	hconf	5' TATGGCAGAAGGGAAGGGGAGC 3'; 5' TCCCTCAGAATACCCAGCAGCC 3	4.1	5' AGGGCTGCTGGGTATTCTGAGG 3'; 5' TGACTCCAGCCCCTCCATTTCC 3'	1
RCC1L / WBSCR16	8	T6	7	74476021	74484051	conf	5' TGGTTCTTTGGAGATGGCGTGG 3'; 5' CGTCAACTGTCTGGGAAGTGGG 3'	4.4	5' TGGTTCTTTGGAGATGGCGTGG 3'; 5' ACACGGGCTGAGAAGTTTCACG 3'	0.4
ALDH16A1	1.6	B10	19	49962668	49964273	hconf	5' CATCCTGCCACCCACATTCTCC 3'; 5' CACTGCTGAGTCATGTGGGAGG 3'	0.6	5' CACTGCTGAGTCATGTGGGAGG 3'; 5' CTTGCTTTCTCGACAGGCTGGC 3'	0.2
HDAC1	1	T1	1	32795177	32796193	conf	5' TGGAAAGGTAGGAGTGGGTGGG 3'; 5' TGAGACCGAGACCACAACTCCC 3'	0.6	5' TGAGACCGAGACCACAACTCCC 3'; 5' GTGTGGTGTATGGGAGGAGTGC 3'	0.2
EGFR	5.7	T8	7	55082513	55088252	conf	5' TGGCAGTGGAAGGAGAAGAGG 3'; 5' CTCCTCGCATTCTCCTCCTCC 3'	1.8	5' CTCCTCGCATTCTCCTCCTCC 3'; 5' TTGGGTTCCTTCCTCAGCTTCC 3'	1
ERBB2 / HER2	15.7	T8 (merged)	17	37850116	37865853	conf	5' TTGTTCTTGTTGCGGGTGTGG 3'; 5' GAAAGAGTTCCTCAGAGTAGGC 3'	4.7	5' CCAAAATAACGCCCTGCTGCC 3'; 5' GGTGTAAAGTGGGAGAGTTCC 3'	4.4
IGLL5	12.4	B6A	22	23223572	23235960	hconf	5' AACTTGCTCCCGTCTCTCAGG 3'; 5' GGAGGTGTCTACAGGGCATCC 3'	1.3	5' GGAGGTGTCTACAGGGCATCC 3'; 5' TGGCTTCCCTTTCTACCAGGC 3'	0.2
MUC4	1	B6C	3	195496359	195497342	conf	5' TCTAATCCAAGACTCGGCCTGC 3'; 5' TACTTGACCTCACAGCAGCCC 3'	2.2	5' TACTTGACCTCACAGCAGCCC 3'; 5' GGACAGAGGAGATGGTGTGGC 3'	0.3
MYH11	2.3	B14C (merged)	16	15831575	15833873	conf	5' TCCTTCCATTCCTGCCACTGG 3'; 5' AGCCCGAAACTCCTAAGCTCC 3'	1.8	5' AGCCCGAAACTCCTAAGCTCC 3'; 5' AGGGTGATTTCGAGAGGTGGC 3'	0.3
SLC12A8	6	B14A	3	124930132	124936159	conf	5' GGTTTCCTGTTTGCTGTCACC 3'; 5' CCAGGTGGATCCTCTCCTACC 3'	3.7	5' CCAGGTGGATCCTCTCCTACC 3'; 5' AAAGGCTGGACAGGACTAGGG 3'	0.7

Supplementary Table 3. Selected eccDNAs and oligos used for outward PCR validation and Sanger sequencing. Seventeen of the selected eccDNAs were validated by both outward PCR and Sanger sequencing. Three recorded eccDNAs did not have final Sanger sequencing validation (*HDAC1*, *EGFR* and *SLC12A8*) and unlike the others, had less coverage and support from structural variation reads, indicating they could be rarer eccDNA types. PCR data suggested that two recorded circles in close proximity at *ERBB2* and *MYH11* should be merged into single elements [*ERBB2*^{circle}] and [*MYH11*^{circle}].

Chr	Start	End	Sample	No. RNA-Seq reads matching eccDNA-junctions	EccDNA length	Chromosome, start, end of detected split-transcript	Length of split-transcript interval	Confidence of RNA split-transcript detection	Annotated gene on eccDNA	Annotated gene on split-transcript interval
**chr1**	565016	565195	04t	2	179	chr1|565019|565188	169	lowq	N.A.	N.A.
**chr1**	565285	565859	07t	2	574	chr1|565301|565432	131	conf	N.A.	N.A.
**chr1**	566131	566324	07t	1	193	chr1|566144|566316	172	lowq	*JA429831*	*JA429831*
**chr1**	566149	566358	04t	6	209	chr1|566157|566276;chr1|566163|566266	119|103	lowq|lowq	*JB137814*	*JB137814|JB137814*
**chr1**	566166	566371	06t	1	205	chr1|566168|566276	108	lowq	*JB137814*	*JB137814*
**chr1**	566593	566749	08t	1	156	chr1|566607|566738	131	conf	N.A.	N.A.
**chr1**	91852963	91853121	08t	2	158	chr1|91852970|91853106	136	lowq	*HFM1*	*HFM1*
**chr13**	110076501	110076651	15t	3	150	chr13|110076516|110076628	112	lowq	N.A.	N.A.
**chr14**	23892824	23893406	05t	6	582	chr14|23892839|23892893	54	lowq	*MYH7*	*MYH7*
**chr14**	23893178	23893294	08t	1	116	chr14|23893181|23893283	102	lowq	*MYH7*	*MYH7*
**chr15**	23292779	23293264	09t	1	485	chr15|23292797|23292845	48	conf	*HERC2P2*	*HERC2P2*
**chr17**	10432003	10439712	07t	8	7709	chr17|10432007|10432084;chr17|10432007|10432071	77|64	hconf|lowq	*AK097500,MYH2*	*AK097500, MYH2|AK097500,MYH2*
**chr18**	192072	192156	03t	1	84	chr18|192074|192156	82	hconf	*USP14*	*USP14*
**chr2**	179391101	180009329	04t	2	618228	chr2|179391105|179425570	34465	lowq	*AK123298,AX746670,CCDC141,MIR548N,SESTD1,TTN,TTN-AS1*	*MIR548N,TTN,TTN-AS1*
**chr2**	179424913	179428233	08t	2	3320	chr2|179424932|179425099	167	lowq	*MIR548N,TTN, TTN-AS1*	*MIR548N,TTN,TTN-AS1*
**chr2**	179587767	179605879	06t	1	18112	chr2|179587773|179587926	153	lowq	*TTN*	*TTN*
**chr2**	179621874	179621956	07t	28	82	chr2|179621875|179621934	59	hconf	*TTN*	*TTN*
**chr2**	179621874	179621956	05t	48	82	chr2|179621875|179621934;chr2|179621888|179621956	59|68	hconf|lowq	*TTN*	*TTN*
**chr20**	57270075	57270395	07t	1	320	chr20|57270088|57270389	301	conf	*NPEPL1*	*NPEPL1*
**chr21**	27909857	27912117	06t	3	2260	chr21|27909873|27911839;chr21|27909873|27911114	1966|1241	conf|conf	*CYYR1*	*CYYR1|CYYR1*
**chr21**	9827315	9827461	04t	4	146	chr21|9827334|9827456	122	hconf	N.A.	N.A.
**chr21**	9827332	9827476	05t	2	144	chr21|9827335|9827440	105	lowq	N.A.	N.A.
**chr5**	79947275	79947406	07t	2	131	chr5|79947290|79947303	13	lowq	*DHFR*	*DHFR*
**chr7**	75353569	75371757	08t	2	18188	chr7|75353570|75371757	18187	hconf	*HIP1*	*HIP1*
**chr8**	70602354	70602511	09t	5	157	chr8|70602355|70602460	105	lowq	*SLCO5A1*	*SLCO5A1*

Supplementary Table 4. List of detected eccDNA transcripts in muscle. Table of 25 detected eccDNAs for which RNA-sequenced split-transcripts matched eccDNA-detected coordinates (n = 16). Columns from left: A) chromosome; B-C) Start and end coordinates; D) Sample with transcribed eccDNA; E) Number of detected split-transcript reads matching eccDNA; F) length of eccDNA; G) Coordinates (chromosome, start, end) of detected split-transcript; H) Length of split-transcript interval; I) Confidence of eccDNA detection (lowq = low confidence, conf = confidence, hconf = high confidence); J) Gene(s) on eccDNA; K) Split-transcript origin gene. Detection of different split-transcripts within eccDNA coordinates is separated with a vertical line in columns G to I. N.A., no annotated genes.

References for supplementary information

1. Cox, D., Yuncken, C. & Spriggs, A. I. MINUTE CHROMATIN BODIES IN MALIGNANT TUMOURS OF CHILDHOOD. *Lancet* **1,** 55–58 (1965).
2. Pauletti, G., Lai, E. & Attardi, G. Early appearance and long-term persistence of the submicroscopic extrachromosomal elements (amplisomes) containing the amplified *DHFR* genes in human cell lines. *Proceedings of the National Academy of Sciences* **87,** 2955–2959 (1990).
3. Turner, K. M. *et al.* Extrachromosomal oncogene amplification drives tumour evolution and genetic heterogeneity. *Nature* **543,** 122–125 (2017).
4. Shore, D., Langowski, J. & Baldwin, R. L. DNA flexibility studied by covalent closure of short fragments into circles. *Proceedings of the National Academy of Sciences* **78,** 4833–4837 (1981).
5. Kiyama, R., Matsui, H., Okumura, K. & Oishi, M. A group of repetitive human DNA families that is characterized by extrachromosomal oligomers and restriction-fragment length polymorphism. *Journal of Molecular Biology* **193,** 591–597 (1987).
6. Carroll, S. M. *et al.* Characterization of an episome produced in hamster cells that amplify a transfected CAD gene at high frequency: functional evidence for a mammalian replication origin. *Molecular and Cellular Biology* **7,** 1740–1750 (1987).
7. Seising, E. & Storb, U. Mapping of immunoglobulin variable region genes: relationship to the 'deletion' model of immunoglobulin gene rearrangement. *Nucleic Acids Research* **9,** 5725–5736 (1981).
8. Shibata, Y. *et al.* Extrachromosomal microDNAs and chromosomal microdeletions in normal tissues. *Science* **336,** 82–86 (2012).
9. Tomaska, L., Nosek, J., Makhov, A. M., Pastorakova, A. & Griffith, J. D. Extragenomic double-stranded DNA circles in yeast with linear mitochondrial genomes: potential involvement in telomere maintenance. *Nucleic Acids Research* **28,** 4479–4487 (2000).
10. Levan, G., Mandahl, N., Bregula, U., Klein, G. & Levan, A. Double minute chromosomes are not centromeric regions of the host chromosomes. *Hereditas* **83,** 83–90 (1976).
11. Guilherme, R. S. *et al.* Mechanisms of ring chromosome formation, ring instability and clinical consequences. *BMC Med. Genet.* **12,** 171 (2011).
12. McGinniss, M. J. *et al.* Mechanisms of ring chromosome formation in 11 cases of human ring chromosome 21. *Am. J. Hum. Genet.* **50,** 15–28 (1992).
13. Smith, C. A. & Vinograd, J. Small polydisperse circular DNA of HeLa cells. *Journal of Molecular Biology* **69,** 163–178 (1972).
14. Cohen, S. & Lavi, S. Induction of circles of heterogeneous sizes in carcinogen-treated cells: two-dimensional gel analysis of circular DNA molecules. *Molecular and Cellular Biology* **16,** 2002–2014 (1996).
15. Rush, M. G. & Misra, R. Extrachromosomal DNA in Eucaryotes. *Plasmid* **14,** 177–191 (1985).
16. Gresham, D. *et al.* Adaptation to diverse nitrogen-limited environments by deletion or extrachromosomal element formation of the *GAP1* locus. *Proceedings of the National Academy of Sciences* **107,** 18551–18556 (2010).

17. Møller, H. D., Parsons, L., Jørgensen, T. S., Botstein, D. & Regenberg, B. Extrachromosomal circular DNA is common in yeast. *Proceedings of the National Academy of Sciences* **112,** E3114–E3122 (2015).
18. Møller, H. D. *et al.* Formation of Extrachromosomal Circular DNA from Long Terminal Repeats of Retrotransposons in *Saccharomyces cerevisiae*. *G3* **6,** 453–462 (2015).
19. Møller, H. D. *et al.* Genome-wide Purification of Extrachromosomal Circular DNA from Eukaryotic Cells. *J Vis Exp* e54239 | (2016). doi:10.3791/54239
