# Supplementary material for: Circular DNA elements of chromosomal origin are common in healthy human somatic tissue
Source: Nat Commun. 2018 Mar 14;9:1069. doi: 10.1038/s41467-018-03369-8 (PMC5852086; doi:10.1038/s41467-018-03369-8)
Supplement: Supplementary file 3 — Description of Additional Supplementary Files [file 41467_2018_3369_MOESM3_ESM.pdf]

Description of Additional Supplementary Files

File Name: Supplementary Data 1
Description: List of detected eccDNAs from muscle samples. Total list of detected eccDNAs from muscle samples of men, age 60-65 (n = 16), containing 138,681 analyzed chromosomal regions including mitochondria. Columns from left: A, sample name; B, chromosome (UCSC convention); C, chromosome (Ensembl convention); D-E, start and end coordinates of recorded eccDNA; F-G, empty columns; H, number of read variants from soft-clipped (SC), discordant paired-end reads (PE) and split reads (SE) supporting a circular structure; I, coverage level upstream, within and downstream of listed coordinates; J, three confidence levels of eccDNA as minimal (lowqual), medium (conf) and highly confident (hconf); K, coding genes within region with • if none (either intergenic, noncoding gene or pseudogene).

File Name: Supplementary Data 2
Description: List of detected eccDNAs from leukocytes. List of 10,228 chromosomal regions recorded as eccDNA from leukocytes of men, age 60-65 (n = 16). Columns from left: A, sample name; B, chromosome (UCSC convention); C, chromosome (Ensembl convention); D-E, start and end coordinates of recorded eccDNA; F-G, empty columns; H, number of read variants from soft-clipped (SC), discordant paired-end reads (PE) and split reads (SE) supporting a circular structure; I, coverage level upstream, within and downstream (of listed coordinates; J, three confidence levels of eccDNA as minimal (lowqual), medium (conf) and highly confident (hconf); K, coding genes within region with • if none (either intergenic, noncoding gene or pseudogene).

File Name: Supplementary Data 3
Description: List of detected eccDNA junctions >25 kb apart. A total list of 1,011 chromosomal-derived eccDNAs, likely larger than 25 kb. List includes eccDNA from all investigated samples (n = 40).

File Name: Supplementary Data 4
Description: Recurrent >25 kb eccDNAs. List of 100 chromosomal regions with detected eccDNA junctions more than 25 kb apart and present in minimum two samples, having a maximum 5 kb differences in the start and end coordinates. List includes detected eccDNAs from all investigated samples (n = 40).

File Name: Supplementary Data 5
Description: EccDNA counts and expression levels for each human gene. Median transcript mRNA levels of human genes and detected eccDNA count per gene. Columns: A, human gene; B, median transcript level in muscle samples (T1-T16, n = 16); C, eccDNA counts per gene (n = 16); D, median transcript level in muscle samples from physically active men and; E, eccDNA counts per gene in the active group (n = 8); F, median transcript level in muscle samples from physically inactive men

(sedentary) and; G, eccDNA counts per gene in the inactive group (n = 8). Genes not included in transcriptome analysis, #NUM!.

File Name: Supplementary Data 6

Description: EccDNA identifications in multiple blood lysates. Table display analyses of 22,800 chromosomal regions where eccDNAs were detected from four blood lysates of participant 6 (B6A-D, n = 4) and four blood lysates participant 14 (B14A-D, n = 4). Columns from left: A, sample name; B, chromosome (UCSC convention); C, chromosome (Ensembl convention); D-E, start and end coordinates of recorded eccDNA; F-G, empty columns; H, number of read variants from soft-clipped (SC), discordant paired-end reads (PE) and split reads (SE) supporting a circular structure; I, coverage level upstream, within and downstream of listed coordinates; J, three confidence levels of eccDNA as minimal (lowqual), medium (conf) and highly confident (hconf); K, coding genes within region with • if none (either intergenic, noncoding gene or pseudogene).
